# Glycinium hydrogen fumarate glycine solvate monohydrate

**DOI:** 10.1107/S1600536809001974

**Published:** 2009-02-04

**Authors:** S. Natarajan, A. Kalyanasundar, J. Suresh, S. A. Martin Britto Dhas, P. L. Nilantha Lakshman

**Affiliations:** a Department of Physics, Madurai Kamaraj University, Madurai 625 021, India; b Department of Physics, The Madura College, Madurai 625 011, India; c Department of Food Science and Technology, Faculty of Agriculture, University of Ruhuna, Mapalana, Kamburupitiya (81100), Sri Lanka

## Abstract

In the title compound, C_2_H_6_NO_2_^+^·C_4_H_3_O_4_^−^·C_2_H_5_NO_2_·H_2_O, the asymmetric unit contains two glycine residues, one protonated and one in the zwitterionic form, a hydrogen fumarate anion and a water mol­ecule. Through N—H⋯O and O—H⋯O hydrogen bonds, mol­ecules assemble in layers parallel to the (10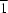) plane, one layer of hydrogen fumarate anions alternating with two layers of glycine mol­ecules. In each glycine layer, hydrogen bonds generate an *R*_4_^4^(19) graph-set motif. Further hydrogen bonds involving the water mol­ecule and the hydrogen fumarate anions result in the formation of a three-dimensional network.

## Related literature

For related structures and general background, see: Alagar *et al.* (2003*a* ▶,*b* ▶); Kvick *et al.* (1980 ▶). For hydrogen-bonding motifs, see: Etter (1990 ▶); Bernstein *et al.* (1994 ▶).
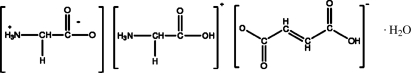


## Experimental

### 

#### Crystal data

C_2_H_6_NO_2_^+^·C_4_H_3_O_4_^−^·C_2_H_5_NO_2_·H_2_O
*M* *_r_* = 284.23
Monoclinic, 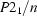

*a* = 13.0580 (12) Å
*b* = 6.8251 (7) Å
*c* = 15.3263 (14) Å
β = 112.65 (2)°
*V* = 1260.6 (3) Å^3^
*Z* = 4
Mo *K*α radiation
μ = 0.14 mm^−1^
*T* = 293 (2) K
0.18 × 0.16 × 0.11 mm

#### Data collection

Nonius MACH3 diffractometer
Absorption correction: ψ scan (North *et al.*, 1968 ▶) *T*_min_ = 0.923, *T*_max_ = 0.953
2742 measured reflections
2219 independent reflections
1922 reflections with *I* > 2σ(*I*)
*R*_int_ = 0.020
2 standard reflections frequency: 60 min intensity decay: none

#### Refinement

*R*[*F* ^2^ > 2σ(*F* ^2^)] = 0.033
*wR*(*F* ^2^) = 0.095
*S* = 1.04
2219 reflections
182 parameters
3 restraints
H atoms treated by a mixture of independent and constrained refinement
Δρ_max_ = 0.18 e Å^−3^
Δρ_min_ = −0.21 e Å^−3^

### 

Data collection: *CAD-4 EXPRESS* (Enraf–Nonius, 1994 ▶); cell refinement: *CAD-4 EXPRESS*; data reduction: *XCAD4* (Harms & Wocadlo, 1996 ▶); program(s) used to solve structure: *SHELXS97* (Sheldrick, 2008 ▶); program(s) used to refine structure: *SHELXL97* (Sheldrick, 2008 ▶); molecular graphics: *ORTEPIII* (Burnett & Johnson, 1996 ▶), *ORTEP-32* for Windows (Farrugia, 1997 ▶) and *PLATON* (Spek, 2003 ▶); software used to prepare material for publication: *SHELXL97*.

## Supplementary Material

Crystal structure: contains datablocks global, I. DOI: 10.1107/S1600536809001974/dn2415sup1.cif

Structure factors: contains datablocks I. DOI: 10.1107/S1600536809001974/dn2415Isup2.hkl

Additional supplementary materials:  crystallographic information; 3D view; checkCIF report

## Figures and Tables

**Fig. 1.**
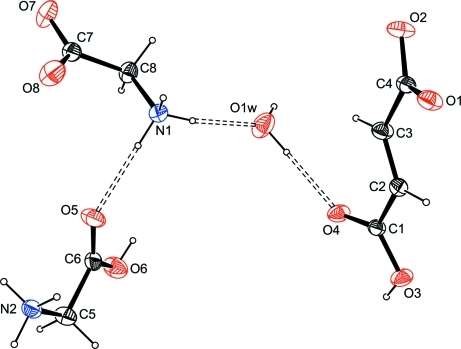
The molecular structure of (I), with the atom-labeling scheme. Displacement ellipsoids are drawn at the 30% probability level. H atoms are represented as small spheres of arbitrary radii. H bonds are shown as dashed lines.

**Fig. 2.**
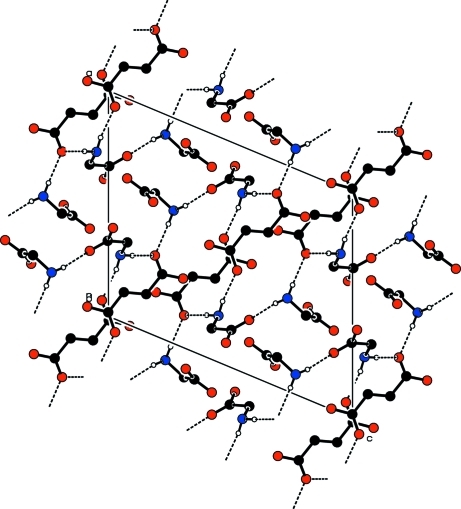
The crystal packing of the molecules down the b axis.

**Fig. 3.**
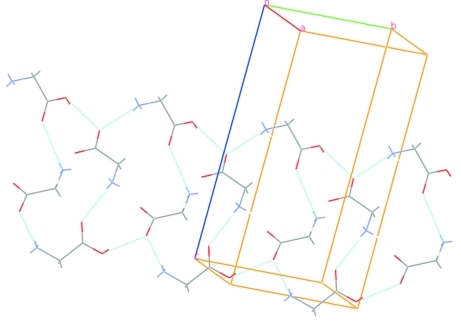
Cyclic chain between the glycine molecules generating a graph set motif R44(19).

## supplementary crystallographic information

### Comment

Glycine is the simplest amino acid and is the only amino acid that is not optically active. This amino acid is essential for the biosynthesis of nucleic acids, as well as the biosynthesis of bile acids, porphyrins, creatine phosphate and other amino acids. Fumaric acid is among the organic compounds widely found in nature, and is key intermediate in the biosynthesis of organic acids. Our main interest in glycine compounds relates to their geometric features of non-covalent interactions at atomic resolution that are important in the structural assembly and function of proteins. X-ray investigations of amino acid complexes with fumaric acid seem to have been first initiated in our laboratory (Alagar *et al.*, (2003*a*), (2003*b*)).

The asymmetric unit is built up from two glycine residues, a ionized fumaric acid and a water molecule linked by hydrogen bonds (Fig. 1). One of the glycine residue has been protonated, and the other one is in the zwitterionic form. The fumaric acid molecule is in the ionized state, as expected from the strength of the acid and the required charge neutrality of the salt.

The glycine carboxyl skeletons including atoms O5, O6, C5, C6 and O7, O8, C7, C8 are both planar  with rms deviations of 0.0025 (6) Å and 0.0002 (6) Å respectively. The N2 and N1 atoms are slightly displaced out of these planes, by 0.138 (3)Å and 0.139 (3)Å respectively, corresponding to a small rotation around C5—C6 and C7—C8 atoms respectively. The relevant torsion angles are O5—C6—C5—N2 of 6.3 (2)°, O6—C6—C5—N2 of -174.47 (13)° and O7—C7—C8—N1 of 174.13 (13)°, O8—C7—C8—N2 of -5.8 (2)°. These can be compared with the corresponding values in pure γ-glycine 167.1 (1)° and -15.4 (1)°, respectively (Kvick *et al.*, (1980)), which is more distorted from planarity. The fumaric acid molecule has a non crystallographic centre of symmetry, and is planar with *trans* configuration about the central C=C bond.

Through N-H···O and O-H···O hydrogen bondings, the molecules assemble in layers parallel to the (1 0 -1) plane, one layer of fumaric acid alternates with two layers of glycine (Fig. 2). In each layer of glycine, the hydrogen bonds generate a graph set motif R_4_^4^(19) (Etter, 1990; Bernstein *et al.*, 1994) (Fig.3, Table 1). Further H bonds involving the water and the fumaric acid result in the formation of a three dimensional network (Fig. 2, Table 1). Unlike the other amino acid fumaric acid complexes (Alagar *et al.*, 2003*a,b* ) there are hydrogen bonds found between the fumaric acid molecules.

### Experimental

Colourless single crystals of the complex were grown, as transparent needles by slow evaporation method from a saturated aqueous solution containing glycine and fumaric acid in 1: 1 stoichiometric ratio.

### Refinement

H atoms attached to C and N atoms were found in difference Fourier but introduced in calculated position and treated as riding on their parent atoms with C-H= 0.97Å (CH_2_) or 0.93Å (aromatic) and N-H= 0.89\%A with *U*_iso_ = 1.2*U*_eq_(C) for CH and *U*_iso_ = 1.5*U*_eq_(N). H atoms of water molecule were located in difference Fourier maps and included in the subsequent refinement using restraints (O-H= 0.85 (1)Å and H···H= 1.39 (2)Å) with U_iso_(H) = 1.5U_eq_(O).

### Crystal data

**Table:**

C_2_H_6_NO_2_^+^·C_4_H_3_O_4_^−^·C_2_H_5_NO_2_·H_2_O	*F*(000) = 600
*M**_r_* = 284.23	*D*_x_ = 1.498 Mg m^−^^3^
Monoclinic, *P*2_1_/*n*	Mo *K*α radiation, λ = 0.71073 Å
Hall symbol: -P 2yn	Cell parameters from 25 reflections
*a* = 13.0580 (12) Å	θ = 2–25°
*b* = 6.8251 (7) Å	µ = 0.14 mm^−^^1^
*c* = 15.3263 (14) Å	*T* = 293 K
β = 112.65 (2)°	Needle, colourless
*V* = 1260.6 (3) Å^3^	0.18 × 0.16 × 0.11 mm
*Z* = 4	

### Data collection

**Table:**

Nonius MACH3 diffractometer	1922 reflections with *I* > 2σ(*I*)
Radiation source: fine-focus sealed tube	*R*_int_ = 0.020
graphite	θ_max_ = 25.0°, θ_min_ = 2.6°
ω–2θ scans	*h* = 0→15
Absorption correction: ψ scan (North *et al.*, 1968)	*k* = −1→8
*T*_min_ = 0.923, *T*_max_ = 0.953	*l* = −18→16
2742 measured reflections	2 standard reflections every 60 min
2219 independent reflections	intensity decay: none

### Refinement

**Table:**

Refinement on *F*^2^	Primary atom site location: structure-invariant direct methods
Least-squares matrix: full	Secondary atom site location: difference Fourier map
*R*[*F*^2^ > 2σ(*F*^2^)] = 0.033	Hydrogen site location: inferred from neighbouring sites
*wR*(*F*^2^) = 0.095	H atoms treated by a mixture of independent and constrained refinement
*S* = 1.04	*w* = 1/[σ^2^(*F*_o_^2^) + (0.0522*P*)^2^ + 0.4133*P*] where *P* = (*F*_o_^2^ + 2*F*_c_^2^)/3
2219 reflections	(Δ/σ)_max_ < 0.001
182 parameters	Δρ_max_ = 0.18 e Å^−^^3^
3 restraints	Δρ_min_ = −0.21 e Å^−^^3^

### Special details

**Table:**

Geometry. All esds (except the esd in the dihedral angle between two l.s. planes) are estimated using the full covariance matrix. The cell esds are taken into account individually in the estimation of esds in distances, angles and torsion angles; correlations between esds in cell parameters are only used when they are defined by crystal symmetry. An approximate (isotropic) treatment of cell esds is used for estimating esds involving l.s. planes.
Refinement. Refinement of *F*^2^ against ALL reflections. The weighted *R*-factor *wR* and goodness of fit *S* are based on *F*^2^, conventional *R*-factors *R* are based on *F*, with *F* set to zero for negative *F*^2^. The threshold expression of *F*^2^ > σ(*F*^2^) is used only for calculating *R*-factors(gt) *etc*. and is not relevant to the choice of reflections for refinement. *R*-factors based on *F*^2^ are statistically about twice as large as those based on *F*, and *R*- factors based on ALL data will be even larger.

### Fractional atomic coordinates and isotropic or equivalent isotropic displacement parameters (Å^2^)

**Table:**

	*x*	*y*	*z*	*U*_iso_*/*U*_eq_	
O1	−0.35547 (8)	0.60201 (18)	0.80983 (7)	0.0389 (3)	
O3	−0.00347 (8)	0.78212 (19)	1.08657 (7)	0.0422 (3)	
H3	0.0631	0.7894	1.1190	0.063*	
O2	−0.30562 (8)	0.70595 (18)	0.69432 (7)	0.0425 (3)	
O5	0.25539 (10)	−0.00488 (18)	0.92332 (8)	0.0484 (3)	
O6	0.39983 (11)	0.16938 (19)	1.01858 (9)	0.0537 (3)	
H6	0.3768	0.2527	0.9770	0.081*	
O7	0.14606 (12)	−0.02415 (18)	0.57652 (9)	0.0534 (3)	
N1	0.08905 (10)	0.19739 (19)	0.76699 (8)	0.0333 (3)	
H1A	0.0202	0.1504	0.7457	0.050*	
H1B	0.0891	0.3205	0.7862	0.050*	
H1C	0.1325	0.1246	0.8152	0.050*	
O8	0.08973 (12)	−0.14584 (19)	0.68336 (9)	0.0559 (4)	
O4	0.05847 (8)	0.72700 (18)	0.97294 (7)	0.0401 (3)	
N2	0.29350 (10)	−0.30064 (19)	1.04930 (9)	0.0338 (3)	
H2A	0.2224	−0.2682	1.0323	0.051*	
H2B	0.3130	−0.3833	1.0979	0.051*	
H2C	0.3033	−0.3578	1.0009	0.051*	
C8	0.13098 (13)	0.1921 (2)	0.69080 (11)	0.0362 (4)	
H8A	0.2082	0.2319	0.7157	0.043*	
H8B	0.0895	0.2846	0.6419	0.043*	
C2	−0.13519 (11)	0.7110 (2)	0.93693 (10)	0.0302 (3)	
H2	−0.1884	0.7059	0.9634	0.036*	
C1	−0.01744 (11)	0.7426 (2)	1.00091 (9)	0.0280 (3)	
C3	−0.16785 (12)	0.6899 (2)	0.84527 (10)	0.0346 (3)	
H3A	−0.1141	0.6923	0.8194	0.041*	
C7	0.12086 (12)	−0.0101 (2)	0.64808 (10)	0.0324 (3)	
C4	−0.28582 (11)	0.6623 (2)	0.77979 (9)	0.0287 (3)	
C6	0.33294 (12)	0.0206 (2)	0.99677 (10)	0.0338 (3)	
C5	0.36250 (13)	−0.1231 (2)	1.07712 (11)	0.0388 (4)	
H5A	0.4401	−0.1591	1.0971	0.047*	
H5B	0.3523	−0.0618	1.1303	0.047*	
O1W	0.07651 (14)	0.58567 (19)	0.80916 (10)	0.0601 (4)	
H1W	0.071 (2)	0.631 (4)	0.8594 (11)	0.090*	
H2W	0.088 (2)	0.682 (3)	0.7788 (15)	0.090*	

### Atomic displacement parameters (Å^2^)

**Table:**

	*U*^11^	*U*^22^	*U*^33^	*U*^12^	*U*^13^	*U*^23^
O1	0.0269 (5)	0.0548 (7)	0.0319 (5)	0.0017 (5)	0.0080 (4)	0.0105 (5)
O3	0.0298 (5)	0.0669 (8)	0.0243 (5)	0.0018 (5)	0.0042 (4)	−0.0097 (5)
O2	0.0319 (6)	0.0647 (8)	0.0241 (5)	−0.0057 (5)	0.0034 (4)	0.0094 (5)
O5	0.0503 (7)	0.0474 (7)	0.0359 (6)	0.0032 (5)	0.0039 (5)	0.0119 (5)
O6	0.0615 (8)	0.0406 (7)	0.0504 (7)	−0.0074 (6)	0.0119 (6)	0.0091 (6)
O7	0.0852 (9)	0.0354 (6)	0.0595 (8)	−0.0111 (6)	0.0498 (7)	−0.0103 (6)
N1	0.0337 (6)	0.0342 (7)	0.0303 (6)	0.0007 (5)	0.0105 (5)	−0.0021 (5)
O8	0.0896 (10)	0.0358 (7)	0.0515 (7)	−0.0176 (6)	0.0374 (7)	−0.0025 (6)
O4	0.0281 (5)	0.0617 (8)	0.0274 (5)	−0.0029 (5)	0.0073 (4)	−0.0041 (5)
N2	0.0350 (6)	0.0359 (7)	0.0314 (6)	0.0050 (5)	0.0137 (5)	0.0077 (5)
C8	0.0411 (8)	0.0313 (8)	0.0408 (9)	−0.0030 (6)	0.0208 (7)	−0.0021 (6)
C2	0.0274 (7)	0.0322 (8)	0.0286 (7)	0.0027 (6)	0.0081 (6)	−0.0002 (6)
C1	0.0304 (7)	0.0261 (7)	0.0236 (7)	0.0020 (6)	0.0062 (6)	0.0007 (5)
C3	0.0261 (7)	0.0472 (9)	0.0278 (7)	0.0007 (6)	0.0074 (6)	0.0036 (6)
C7	0.0327 (7)	0.0300 (8)	0.0332 (8)	−0.0023 (6)	0.0115 (6)	0.0001 (6)
C4	0.0267 (7)	0.0317 (7)	0.0245 (7)	0.0028 (6)	0.0062 (6)	0.0031 (6)
C6	0.0356 (8)	0.0343 (8)	0.0340 (8)	0.0081 (6)	0.0161 (7)	0.0030 (6)
C5	0.0367 (8)	0.0410 (9)	0.0333 (8)	0.0007 (7)	0.0074 (6)	0.0067 (7)
O1W	0.1029 (11)	0.0375 (7)	0.0570 (8)	−0.0019 (7)	0.0496 (8)	−0.0013 (6)

### Geometric parameters (Å, °)

**Table:**

O1—C4	1.2375 (17)	N2—H2B	0.8900
O3—C1	1.2826 (17)	N2—H2C	0.8900
O3—H3	0.8200	C8—C7	1.511 (2)
O2—C4	1.2691 (17)	C8—H8A	0.9700
O5—C6	1.2021 (19)	C8—H8B	0.9700
O6—C6	1.296 (2)	C2—C3	1.309 (2)
O6—H6	0.8200	C2—C1	1.4869 (19)
O7—C7	1.2643 (19)	C2—H2	0.9300
N1—C8	1.4686 (19)	C3—C4	1.4917 (19)
N1—H1A	0.8900	C3—H3A	0.9300
N1—H1B	0.8900	C6—C5	1.504 (2)
N1—H1C	0.8900	C5—H5A	0.9700
O8—C7	1.2185 (19)	C5—H5B	0.9700
O4—C1	1.2267 (18)	O1W—H1W	0.858 (10)
N2—C5	1.472 (2)	O1W—H2W	0.852 (10)
N2—H2A	0.8900		
			
C1—O3—H3	109.5	O4—C1—O3	124.08 (13)
C6—O6—H6	109.5	O4—C1—C2	121.76 (12)
C8—N1—H1A	109.5	O3—C1—C2	114.14 (12)
C8—N1—H1B	109.5	C2—C3—C4	124.12 (14)
H1A—N1—H1B	109.5	C2—C3—H3A	117.9
C8—N1—H1C	109.5	C4—C3—H3A	117.9
H1A—N1—H1C	109.5	O8—C7—O7	124.76 (15)
H1B—N1—H1C	109.5	O8—C7—C8	119.37 (13)
C5—N2—H2A	109.5	O7—C7—C8	115.87 (13)
C5—N2—H2B	109.5	O1—C4—O2	125.13 (13)
H2A—N2—H2B	109.5	O1—C4—C3	120.55 (12)
C5—N2—H2C	109.5	O2—C4—C3	114.32 (13)
H2A—N2—H2C	109.5	O5—C6—O6	126.57 (15)
H2B—N2—H2C	109.5	O5—C6—C5	122.03 (15)
N1—C8—C7	111.70 (12)	O6—C6—C5	111.39 (13)
N1—C8—H8A	109.3	N2—C5—C6	111.37 (12)
C7—C8—H8A	109.3	N2—C5—H5A	109.4
N1—C8—H8B	109.3	C6—C5—H5A	109.4
C7—C8—H8B	109.3	N2—C5—H5B	109.4
H8A—C8—H8B	107.9	C6—C5—H5B	109.4
C3—C2—C1	123.39 (14)	H5A—C5—H5B	108.0
C3—C2—H2	118.3	H1W—O1W—H2W	107.4 (19)
C1—C2—H2	118.3		
			
O5—C6—C5—N2	6.3 (2)	O7—C7—C8—N1	174.08 (13)
O6—C6—C5—N2	−174.52 (13)	O8—C7—C8—N1	−5.8 (2)

### Hydrogen-bond geometry (Å, °)

**Table:**

*D*—H···*A*	*D*—H	H···*A*	*D*···*A*	*D*—H···*A*
O3—H3···O2^i^	0.82	1.66	2.4743 (15)	174
O6—H6···O7^ii^	0.82	1.70	2.4871 (17)	160
N1—H1A···O1^iii^	0.89	2.01	2.8891 (17)	168
N1—H1B···O1W	0.89	1.86	2.7475 (19)	172
N1—H1C···O5	0.89	2.01	2.8907 (18)	168
N2—H2A···O4^iv^	0.89	1.98	2.8386 (16)	162
N2—H2B···O1^v^	0.89	1.98	2.8638 (17)	170
N2—H2C···O7^vi^	0.89	1.93	2.8000 (17)	164
O1W—H1W···O4	0.86 (1)	1.93 (1)	2.7825 (17)	178 (2)
O1W—H2W···O8^vii^	0.85 (1)	1.88 (1)	2.7133 (18)	165 (2)

Symmetry codes: (i) *x*+1/2, −*y*+3/2, *z*+1/2; (ii) −*x*+1/2, *y*+1/2, −*z*+3/2; (iii) −*x*−1/2, *y*−1/2, −*z*+3/2; (iv) *x*, *y*−1, *z*; (v) −*x*, −*y*, −*z*+2; (vi) −*x*+1/2, *y*−1/2, −*z*+3/2; (vii) *x*, *y*+1, *z*.
